# Effects of Enrofloxacin on the Epiphytic Algal Communities Growing on the Leaf Surface of *Vallisneria natans*

**DOI:** 10.3390/antibiotics11081020

**Published:** 2022-07-29

**Authors:** Qi Chen, Luqi Jin, Yuan Zhong, Gaohua Ji

**Affiliations:** 1 National Demonstration Center for Experimental Fisheries Science Education, Shanghai Ocean University, Shanghai 201306, China; aqua_qchen@163.com (Q.C.); zhongyuan9922@163.com (Y.Z.); 2 Engineering Research Center of Environmental DNA and Ecological Water Health Assessment, Shanghai Ocean University, Shanghai 201306, China; j743769395@126.com

**Keywords:** enrofloxacin, *Vallisneria natans*, epiphytic algae, antibiotics

## Abstract

Enrofloxacin (ENR) is a member of quinolones, which are extensively used in livestock farming and aquaculture to fight various bacterial diseases, but its residues are partially transferred to surface water and affect the local aquatic ecosystem. There are many studies on the effect of ENR on the growth of a single aquatic species, but few on the level of the aquatic community. Epiphytic algae, which are organisms attached to the surface of submerged plants, play an important role in the absorption of nitrogen and phosphorus in the ecological purification pond which are mainly constructed by submerged plants, and are commonly used in aquaculture effluent treatment. Enrofloxacin (ENR) is frequently detected in aquaculture ponds and possibly discharged into the purification pond, thus imposing stress on the pond ecosystem. Here, we performed a microcosm experiment to evaluate the short-term effects of pulsed ENR in different concentrations on the epiphytic algal communities growing on *Vallisneria natans*. Our results showed an overall pattern of “low-dose-promotion and high-dose-inhibition”, which means under low and median ENR concentrations, the epiphytic algal biomass was promoted, while under high ENR concentrations, the biomass was inhibited. This pattern was mainly attributed to the high tolerance of filamentous green algae and yellow-green algae to ENR. Very low concentrations of ENR also favored the growth of diatoms and cyanobacteria. These results demonstrate a significant alteration of epiphytic algal communities by ENR and also spark further research on the potential use of filamentous green algae for the removal of ENR in contaminated waters because of its high tolerance.

## 1. Introduction

Enrofloxacin (ENR) is a representative of third-generation quinolones. It is often used in livestock farming and aquaculture to treat animal skin infections, bacterial diseases, and respiratory tract mycoplasma infections due to its broad spectrum of antibacterial activity, low cost, and lack of cross-resistance [1,2,3,4]. It has a long half-life in the water environment, and its metabolite, ciprofloxacin, degrades slowly, which can prolong the metabolic time of enrofloxacin in water [5]. This leads to the long-term exposure of aquatic organisms to low concentrations of ENR [6], enhancing their resistance to ENR and producing resistance genes [7,8], and creating chronic toxic effects in the antioxidant defense system and organ and tissue damage [9]. Furthermore, residual ENR endangers people’s health, reduces food safety, and seriously affects the ecological balance of water bodies through food chain transmission [10]. Therefore, ENR has become one of the most frequently detected emerging pollutants in surface water [11,12,13,14].

Aquatic plants, especially submerged plants, play important roles in the function of ecological purification ponds and are widely used in aquaculture effluent treatments [15,16]. Submerged plants degrade pollutants through the absorption and enrichment of them in the water body and the microbial communities formed around the plants [17]. Epiphytic algae are often attached to the surface of submerged plants. Together with bacteria, fungi, protozoa, and inorganic and organic debris, they make up a tiny ecosystem independent of plants and water [18,19] and make a significant contribution to the absorption and degradation of nutrients. Meanwhile, epiphytic algae have a high sensitivity to water pollutants and are closely related to the pH and nutrients of the surrounding environment [20,21,22], making them a useful indicator for monitoring polluted water [23,24]. 

Many studies have evaluated the impact of antibiotics on single species of aquatic organisms, and the non-observed effect concentration varies widely depending on the species [1,25,26], but little has been known of the influence of ENR on aquatic systems at the community level thus far. Therefore, we performed mesocosm experiments to assess the short-term effects of pulsed ENR in different concentrations on the epiphytic algal communities growing on *Vallisneria natans*, a submerged plant that is the most used in ecological restoration and purification. We monitored the cell density and biomass of different groups of algae to evaluate the community alteration under the stress of different concentrations of enrofloxacin (ENR). We hope the outcomes add crucial information to help perform exposure assessments and remove antibiotics from contaminated waters.

## 2. Results

### 2.1. Total Algal Density and Biomass

**Figure 1:**
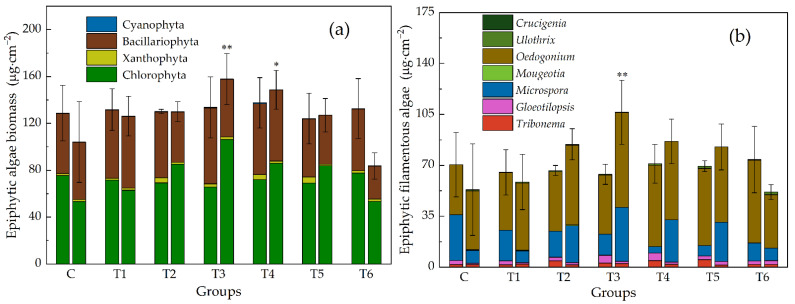
Biomass of epiphytic algae, (**a**) at phylum level, (**b**) epiphytic filamentous algae at genus level. Each group has two histograms (right: Initial; left: 7 d). Groups: C, control group; T1–T6, treatment groups, and the corresponding concentrations of ENR are 0.001, 0.2, 1.13, 6.33, 35.6, and 200 mg · L^−1^, respectively. Stars above the histogram show significant differences between control group and treatment groups (* *p* < 0.05, ** *p* < 0.01).

**Figure 2:**
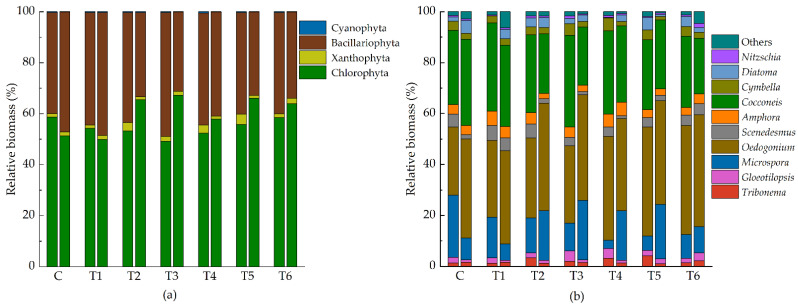
Community composition of relative biomass of epiphytic algae ((**a**) at phylum level, (**b**) at genus level). Each group has two histograms (right: Initial, left: 7 d). Abbreviations for groups are the same as in Figure 1.

The density and biomass changed roughly synchronously. They were both greater than the control group under low and median ENR concentrations and smaller than the control group under high ENR concentrations, i.e., they exhibited a pattern of “low-dose-promotion and high-dose-inhibition”. The biomass of T3 had the highest value. However, the differences between groups are generally not significant due to the large deviation (Figure 1 and Figure 2). The biomass is more comparable because the cell size varies a lot, so we used biomass in the flowing analysis. A significant relationship was noted between the biomass and ENR concentrations (*p* < 0.05).

### 2.2. Change in Algal Groups

**Figure 3:**
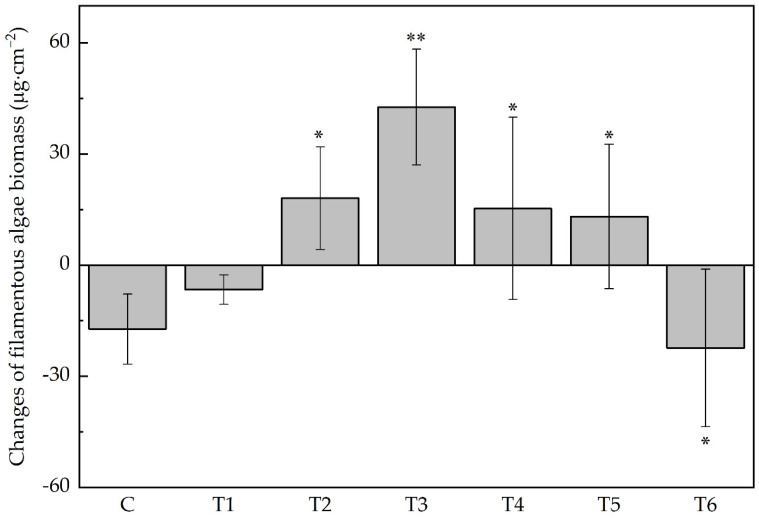
Changes in the filamentous algal biomass. Abbreviations for groups are the same as in Figure 1. Stars above the histogram show significant difference between control group and treatment groups (* *p* < 0.05, ** *p* < 0.01).

The biomass of four main groups of algae in this experiment—Chlorophyta, Xanthophyta, Bacillariophyta, and Cyanophyta—changed asynchronously (Figure 1 and Figure 2). Under low ERN concentrations, they were all greater than the control group, but when ENR exceeded 1.13 mg · L^−1^, diatoms and cyanobacteria decreased along with the increased ENR. In contrast, Chlorophyta and Xanthophyta peaked in T3, and in T5, the recorded biomass was still greater than in the control group. The increase in total biomass was mainly a result of the increase in filamentous algae (Figure 3). The change in the filamentous algal biomass was significantly correlated with the concentration of ENR.

### 2.3. Change of Species

A total of 49 species of epiphytic algae were identified, belonging to 4 phyla and 24 genera (Supplementary Figure S1). Green algae (Chlorophyta) and diatoms (Bacillariophyta) are dominant in the epiphytic algal community, while the relative biomass of the yellow-green algae (Xanthophyta) and blue-green algae (Cyanophyta) was smaller in comparison (Figure 2). 

**Figure 4:**
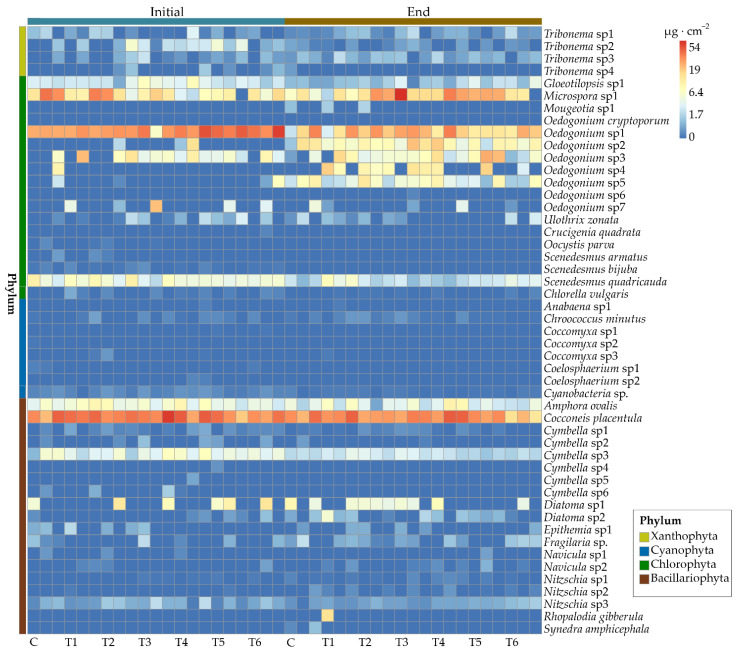
Heatmap of epiphytic algal species biomass. The color of the square indicates the level of the biomass. Each group has three replications. Abbreviations for groups are the same as in Figure 1.

At the end of the experiment, the dominant species slightly varied in the experimental groups (Figure 4). *Cocconeis placentula*, *Microspora* sp1, and *Oedocladium* spp. were commonly dominant in all the samples. In the control group, the higher or highest recorded biomass was for the species *Oedocladium* sp1, *Oedocladium* sp2, *Oedocladium* sp5, *Microspora* sp1, and *C. placentula*. In treatment group T1, the highest values were recorded for the species *Oedocladium* sp1, *Oedocladium* sp4, *Oedocladium* sp3, *C. placentula*, etc. In treatment group T2, the highest values were recorded for the species *Oedocladium* sp1, *Oedocladium* sp2, *Oedocladium* sp4, *Oedocladium* sp5 *Microspora* sp1, and *C. placentula*. *Microspora* sp1, *C. placentula*, *Oedogonium* sp1, *Oedogonium* sp2, and *Oedogonium* sp4 had the highest recorded biomass in treatment group T3. The biomass of *C. placentula*, *Oedogonium* sp1, *Oedogonium* sp2, and *Oedogonium* sp3 was dominant in terms of the biomass in treatment groups T4 and T5. In treatment group T6, the biomass of *C. placentula*, *Oedogonium* sp1, *Oedogonium* sp2, and *Gloeotilopsis* sp1 was relatively high. The relative biomass of *Scenedesmus quadricauda* under the ENR concentration of 200 mg∙L^−1^ was also high.

### 2.4. Community Diversity

**Figure 5:**
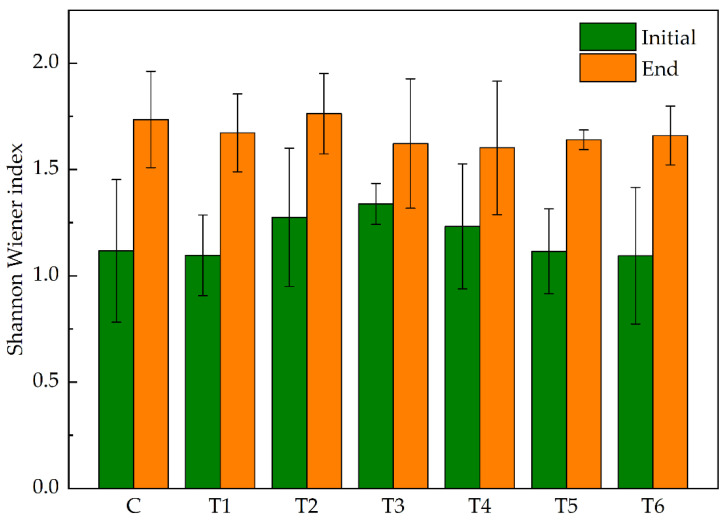
Shannon Wiener index of epiphytic algae at the initial and end of the experiment. Abbreviations for groups are the same as in Figure 1.

Overall, the variation range of the epiphytic algal diversity measured by the Shannon index was 1.21–1.74 (Figure 5). Compared with control group C, treatment group T4 had the lowest Shannon diversity index while treatment group T2 had the highest, but the difference was not significant (*p* > 0.05). 

**Figure 6:**
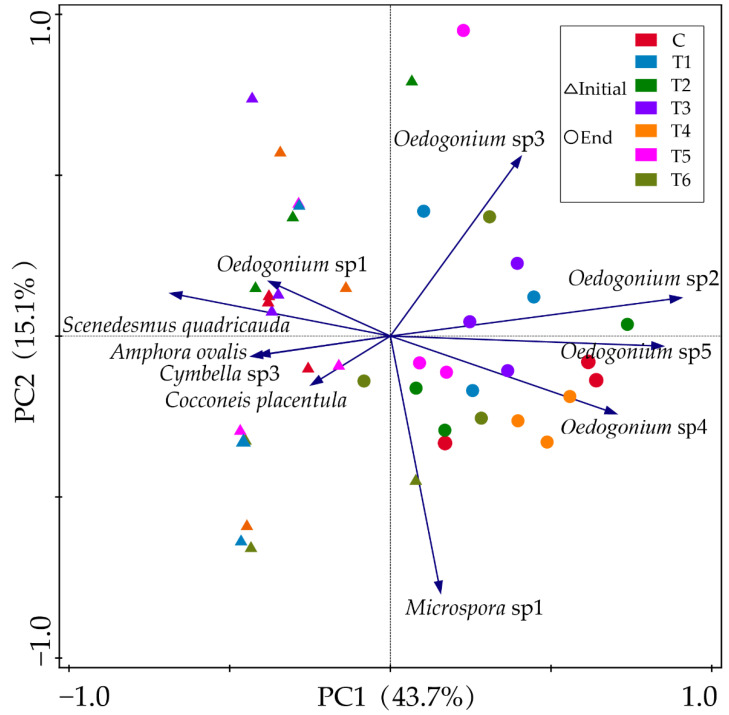
PCA plots of main epiphytic algal species at the initial (triangles) and end (circles) of the experiment. Colors represent different groups. Abbreviations for groups are the same as in Figure 1.

PCA plot revealed the responses of the main epiphytic algal species (relative biomass > 10%) on the surface of *V. natans* to different concentrations of ENR (Figure 6). Overall, the green algal species, *Oedocladium* spp. and *Microspora* sp1, occurred more at the end of experiments in the treatment groups. They gained their highest biomass under moderate ENR concentrations and decreased under the highest concentrations of ENR. 

## 3. Discussion

Different algal species responded differently to antibiotics depending on their cell structure and evolutionary status. Our results showed that ENR, when in concentrations less than 0.2 mg∙L^−1^, stimulated most of the algae to demonstrate growth, while cyanobacteria and diatoms were inhibited when the ENR concentration was higher than 1.13 mg∙L^−1^. When the ENR concentration was less than 6.33 mg∙L^−1^, the dominant species in the treatment groups were mainly *Oedocladium* sp1, *Oedocladium* sp2, and *C. placentula*, and the Cyanophyta and Bacillariophyta species, such as *Chroococcus minutus*, also grew well. However, with the increase in the ENR concentration, when it was higher than 6.33 mg·L^−1^, the dominant species changed to *Microspora* sp1, *Oedocladium* sp1, and *Oedocladium* sp3, and the Cyanophyta almost disappeared. Additionally, according to the PCA analysis, ENR concentrations had less of a negative effect on the density of filamentous algae, such as *Oedocladium* and *Gloeotilopsis*, which means that smaller epiphytic algae exhibit higher sensitivity when contaminated with antibiotics. Smaller cell groups of algae are conducive to increasing the surface-to-volume ratio, improving the absorption efficiency of nutrients, and promoting growth [27]. 

Studies have shown that *Scenedesmus* is a typical alga with an inducible defense ability, and *S. quadricauda* can induce the degradation of antibiotics, such as ciprofloxacin and levofloxacin, through biotransformation or biocatalysis by means of decarboxylation and demethylation [28,29], so the relative abundance of *S. quadricauda* in the ENR concentration of the 200 mg∙L^−1^ treatment group is relatively high compared with the other treatment groups.

The cyanobacterial biomass decreased significantly in most of the treated groups. It has been shown that the cellular structure and metabolic pathways of cyanobacteria are similar to those of bacteria, and most antibiotics can find the corresponding sites of action in cyanobacterial cells, which leads to cyanobacteria’s high sensitivity to antibiotics [30]. On the contrary, there are fundamental differences between green algae and prokaryotes in the cellular structure and composition, so green algae have a low sensitivity to antibiotics [31]. This can explain why Chlorophyta was still the dominant population under different concentrations of ENR and contributed a lot to the total algal biomass. Consequently, the biomass of Chlorophyta determined the changes in the total biomass in each treated group.

There was a pattern of “low-dose-promotion and high-dose-inhibition” because the low concentration of ENR stimulated the increase in the intracellular reactive oxygen of algal cells and consequently stimulated a series of physiological reactions, which had a promoting effect on the growth of epiphytic algae. However, as the ENR concentration continued to increase, the growth inhibition effect of epiphytic algae was more obvious after being stressed to a certain extent [32], and as a whole, the biomass of epiphytic algae rose first and then declined with the increased concentration of ENR. ENR has excitatory toxicity (Hormesis) on the epiphytic algae, which refers to the dose–response relationship in which an agent stimulates the growth of the test species at low doses and inhibits its growth at higher doses [33], that is, the toxic effect of low concentrations of ENR on epiphytic algae is small, which can slightly benefit the growth and reproduction of epiphytic algae [34]. On the contrary, high concentrations of ENR obviously inhibited epiphytic algae, which is not conducive to the growth and reproduction of attached algae. Since ENR promotes algal growth at low concentrations, in the results of the present study, the inflection points of this effect may be depended on a threshold of 1.13 mg∙L^−1^ for Cyanophyta and above 35.6 for filamentous Chlorophyta. Under the threshold, ENR slightly promoted the total biomass and total density of epiphytic algae, while above the threshold, ENR inhibited growth.

It has been suggested that antibiotics affect the algae by affecting the photosynthesis of algae and reducing chlorophyll content, thereby affecting the biomass of algae [35]. For autotrophic algae, chlorophyll plays an important role in the absorption, transmission, and conversion of light energy, and changes in its content directly affect whether algal photosynthesis can be carried out normally, which, in turn, affects the synthesis of organic matter (such as proteins) in algal cells [36]. It has also been found that, on the one hand, ciprofloxacin, ENR, and ofloxacin all affect the photosynthetic pigment content in *Scenedesmus obliquus*, and as the concentration of antibiotics increases, the color of the algae becomes lighter, and the photosynthetic pigment content gradually decreases. On the other hand, when subjected to antibiotic stress, the antioxidant system of the algal cells is affected, which induces a large number of free radicals and a sharp increase in their accumulation in the algal cells. In turn, this causes oxidative stress in the cell and produces peroxidation reactions in the membrane lipids [37], breaking the cell membrane, increasing the permeability of the algal cell membrane, reacting with macromolecular substances in the cell, and affecting the normal metabolic function of the cell [38], and when the ambient antibiotic concentration exceeds the threshold, the antioxidant system of the algal cells can no longer maintain the homeostatic balance of free radical production and scavenging, so oxidative damage occurs, and the algal cells die. Furthermore, antibiotics also affect the transportation of some algal proteins, the replication and transcription of enzyme genes, and the encoding of photosynthetic genes [39].

The growth or culture of microalga-based technology is a potential method for antibiotic removal. Microalgae are autotrophic organisms that can use light energy to grow and can tolerate higher concentrations of antibiotics compared with bacteria [40]. They can make full use of nitrogen, phosphorus, and small organic particles in wastewater. This method can reduce the supply of nutrients in wastewater treatment, and the treated algal biomass can be extracted to obtain valuable by-products, forming a virtuous cycle that has attracted the attention of researchers. A large number of studies have shown that microalgae can effectively remove pollutants from municipal wastewater, industrial wastewater, surface water, domestic wastewater, aquaculture effluents, and medical wastewater [41,42,43,44,45]. Microalgae are not the target organism of antibiotics. A certain concentration of antibiotics has a low effect on the growth and reproduction of microalgae [46]. Our results showed that antibiotics could promote the growth of algae in the presence of low concentrations, and high concentrations of antibiotics may also seriously inhibit the growth of algae, which is consistent with another study [47]. ENR can be removed by micro-green algae, *Chlorella vulgaris* and *Scenedesmus obliquus* [48]. The filamentous algae have a high tolerance to antibiotics, sparking further research on whether they can be used to remove antibiotics.

## 4. Materials and Methods

### 4.1. Cultivation of Epiphytic Algae

*Vallisneria natans* was used as the substrate of epiphytic algae. The plants were collected in a wild pond. We selected plants in good growing conditions and then cleaned and removed the withered and rotten leaves. However, we kept epiphytic algae on the leaves and cultured them in 72 L glass tanks with transparent glass beads in the bottom to fix the roots. The planting density in each tank was about 1200 plants∙m^−2^, and the corresponding weight was 390.4 ± 1.5 g. This density was similar to a wild pond. In order to make the photoperiod and diurnal temperature fluctuation close to natural water conditions, the glass tank was placed in a greenhouse with a shading net. Temperature and light were monitored every day to prevent the adverse effects of intense light and high temperatures. An optical and temperature recorder MX2202 (Onset HOBO, Bourne, MA, USA) was used to record the light intensity at the top of *V. natans* and water temperature, and the underwater quantum meter MQ-210 (Apogee Instruments, Inc., Logan, UT, USA) was used to measure the photosynthetically active radiation at noon.

During the cultivation periods, the water temperature was 26 ± 2.5 °C, and the maximum photosynthetically active radiation on the water surface did not exceed the light saturation point of *V. natans* by 200 μmol∙m^−2^∙s^−1^. All the tanks were connected and circulated by water pipes to ensure that the initial biological community and water quality of each tank were homogeneous. After two weeks of culture, *V. natans* grew well, and epiphytic algae on the leaves were visible to the naked eye.

### 4.2. Experimental Design

The experiment included a control group (C) and six treatment groups (T1~T6), with three replicates in each group. According to the common containment concentration, water solubility, and minimum effective concentration of various aquatic organisms of ENR [1,49], the ENR concentrations in T1~T6 were set as 0.001, 0.2, 1.13, 6.33, 35.6, and 200 mg∙L^−1^, respectively. ENR was dissolved in the culture solution of *V. natans* to make a stock solution. 

Before the addition of ENR, TN and TP concentrations in the tanks were adjusted to 0.16 mg∙L^−1^ and 4 mg∙L^−1^, respectively, simulating the nitrogen and phosphorus concentration in aquaculture ponds and also providing nutrients for plant growth during the experiment. Distilled water was supplemented regularly without changing water to ensure that the volume of water in each tank was 55 L. ENR was added once at the beginning of the experiment to simulate the pulse of aquaculture effluent.

### 4.3. Epiphytic Algal Sampling and Measurement

Before and 7 days after the addition of ENR, 15 *V. natans* were randomly selected from each tank, the leaf surface was carefully cleaned with a soft brush, and the total leaf surface area was recorded. The solution with scrubbed algae was collected and fixed to a constant volume. Then, a 50 mL subsample was taken, and we added 0.75 mL of Lugol’s iodine solution and 2 mL of formalin and sedimented it for more than 96 h. After the algae were completely settled, discarding the supernatant, they were concentrated to a volume of 25 mL. 

For unicell or small colony algal species, we used a 0.1 mL counting chamber (20 mm × 20 mm), counted the samples under the optical microscope (Nikon CX21, Nikon, Tokyo, Japan) with a magnification of 400×, and counted more than 400 algal cells per sample.

For the filamentous algae, it was easy to make a large deviation since the 0.1 mL counting chamber is too small, and the measurement error is unacceptable according to our practice. Therefore, we use a 1 mL counting chamber to count and identify the filamentous algae. Counting was completed with a magnification of 200×, and more than 400 filamentous algal cells per sample were recorded. A total of more than 40,000 cells was observed during the experiment. Algal taxa were identified according to a series of books on freshwater algal flora and an online database [50,51,52,53,54,55,56].

To calculate the biomass of algae, 30 cells of each species were randomly selected and measured. Then, the cell volumes were calculated based on the most approximate geometric shapes. Fresh biomass (wet weight) was estimated by assuming the specific gravity of algal cells of 1 (10^6^ µm^3^ is roughly equivalent to 1 µg fresh algal weight). 

### 4.4. Statistical Analyses

According to the counting results, the cell density and fresh biomass of epiphytic algae (cells · cm^−2^, μg · cm^−2^) per unit area of *V. natans* leaves were calculated. 

In the present study, species with a relative abundance of *Pi* > 10% were considered the dominant species in the community. We used Shannon–Wiener index (*H*) to analyze species diversity characteristics of the epiphyte community by the following formula:

(1) $$H=-\sum_{i=1}^{S}P_{i}\ln P_{i}$$

where $P_{i}$ is the proportion of the entire community made up of species *I*, and *S* is the total number of unique species.

At the significance level of *p* < 0.05, one-way ANOVA was used to compare the differences between six treatment groups (T1~T6) and one control group (C). The vegan package in R3.2.5 was used for PCA analysis and heat map illustrating to compare the similarity and differences of algal community structure.

## 5. Conclusions

The effects of different concentrations of enrofloxacin on the epitope community are different, showing the pattern of “low-dose-promotion and high-dose-inhibition”, but the thresholds are different in regard to different algal groups and species. The tolerance of filamentous Chlorophyta to ENR in epiphytic algae is higher than that of Bacillariophyta and Cyanophyta, sparking further research on the potential use of filamentous green algae for the removal of ENR in contaminated waters because of its high tolerance.

## Data Availability

The data presented in this study are available on request from the corresponding author.

## Supplementary Materials

The following supporting information can be downloaded at: https://www.mdpi.com/article/10.3390/antibiotics11081020/s1, Figure S1: epiphytic algae observed in this study.
