# Supplementary material for: Effects of Enrofloxacin on the Epiphytic Algal Communities Growing on the Leaf Surface of Vallisneria natans
Source: Antibiotics (Basel). 2022 Jul 29;11(8):1020. doi: 10.3390/antibiotics11081020 (PMC9404838; doi:10.3390/antibiotics11081020)
Supplement: Supplementary file 1 [file antibiotics-11-01020-s001.zip › Figure S1(continued).pdf]

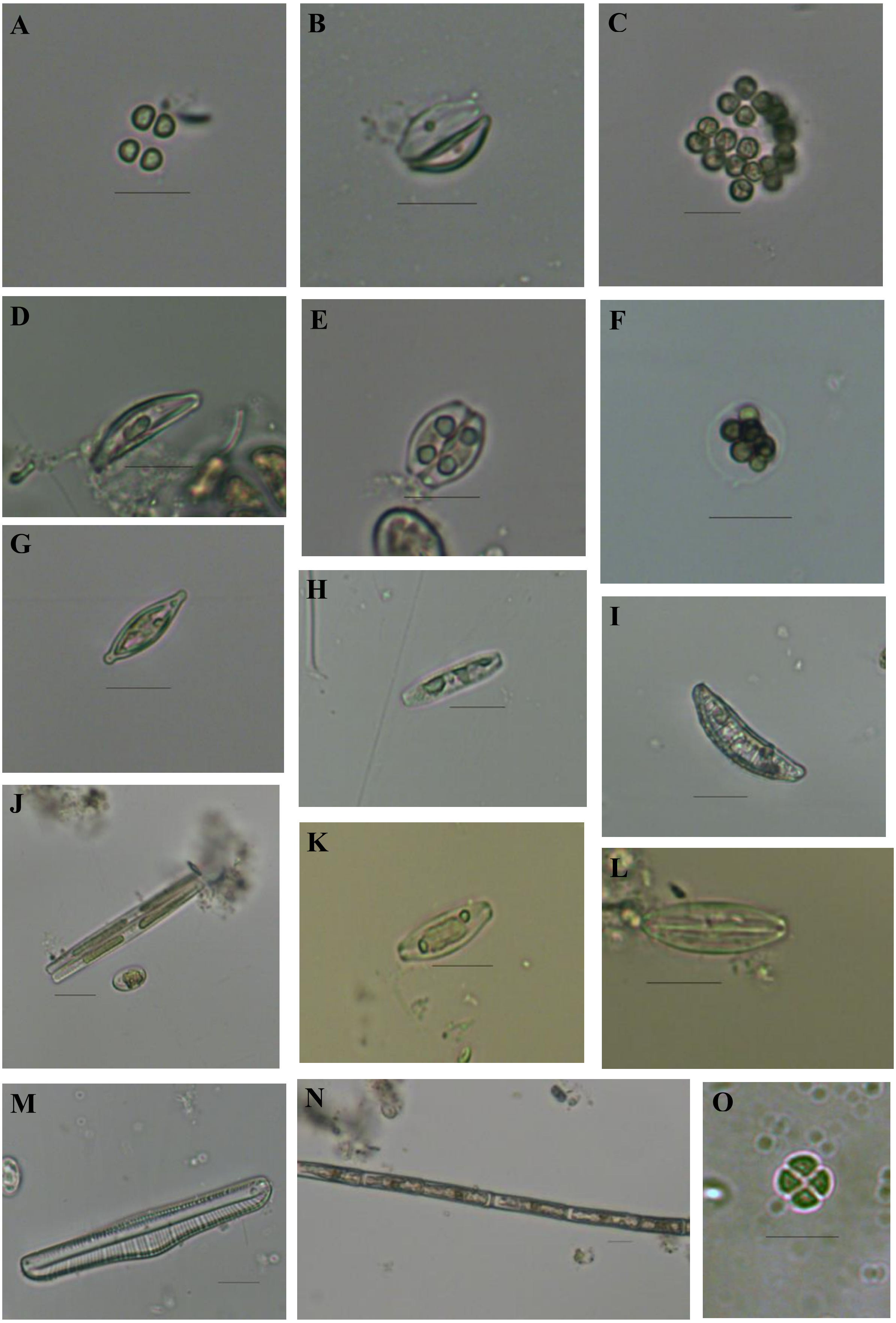

Figure S1 (continued). Epiphytic algae observed in this study. (Scale bars: 20 μm)

A.*Chroococcus*, B. *Cymbella*, C. *Gloeocapsa*, D. *Cymbella*, E. *Cymbella*, F. *Gloeocapsa*, G. *Navicula*, H. *Navicula*, I. *Epithemia*, J. *Synedra*, K. *Cymbella*, L. *Amphora* , M. *Rhopalodia*, N. *Tribonema*, O. *Crucigenia.*
