# Supplementary material for: Effects of Enrofloxacin on the Epiphytic Algal Communities Growing on the Leaf Surface of Vallisneria natans
Source: Antibiotics (Basel). 2022 Jul 29;11(8):1020. doi: 10.3390/antibiotics11081020 (PMC9404838; doi:10.3390/antibiotics11081020)
Supplement: Supplementary file 1 [file antibiotics-11-01020-s001.zip › Figure S1.pdf]

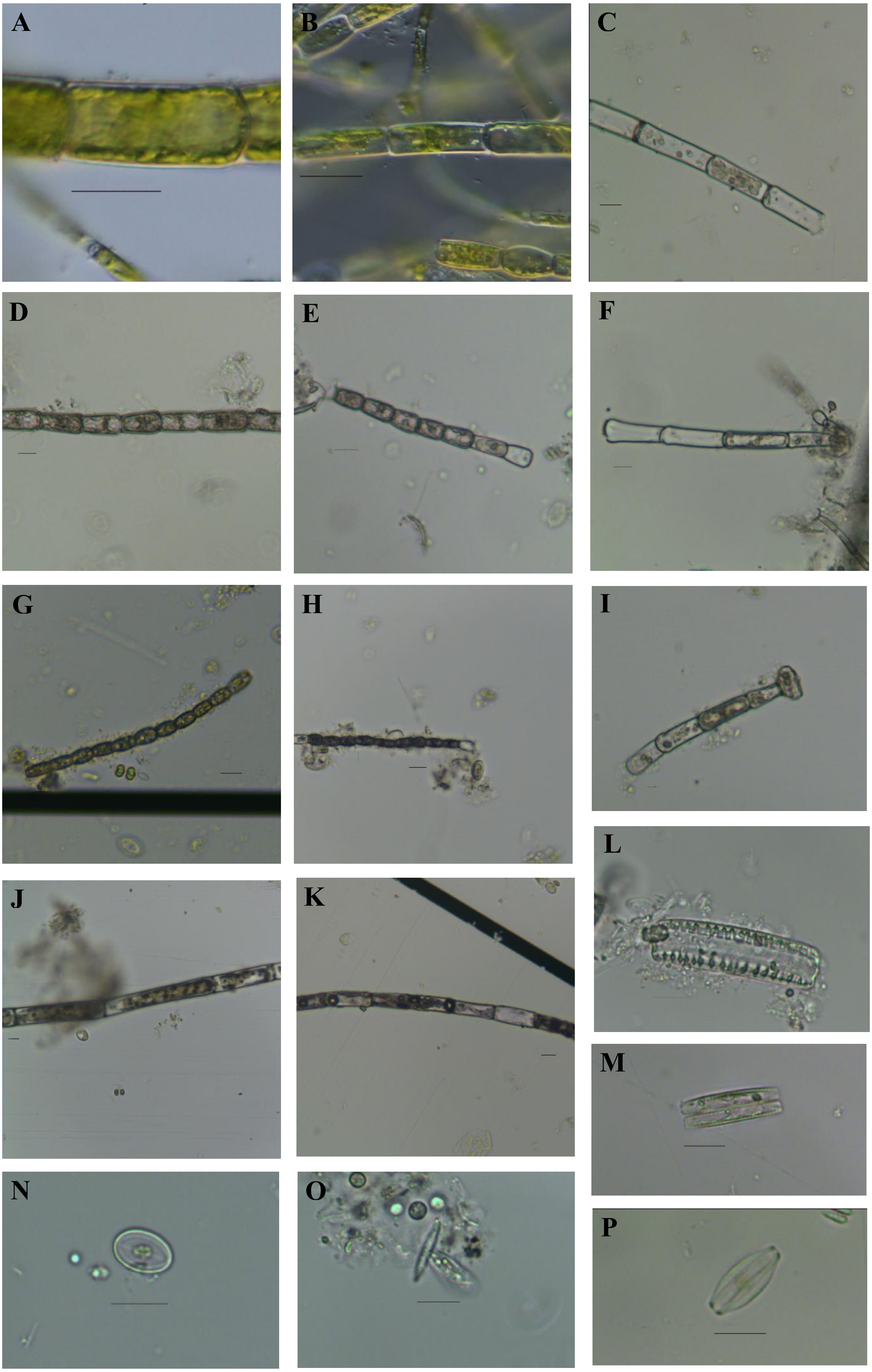

Figure S1. Epiphytic algae observed in this study. (Scale bars: 20 μm)

A. *Microspora*, B. *Gloeotilopsis*, C. *Oedocladium*, D, E. *Ulothrix*, F. *Oedocladium*, G, H. *Tribonema*, I, J, K. *Oedocladium*, L. *Diatoma*, M. *Fragilaria*, N. *Cocconeis*, O. *Nitzschia*, P. *Amphora*
